# Molecular subtypes associated with lymph node metastasis in colorectal cancer reveal the tumor-promoting role of GDI1 and the preclinical activity of deslanoside

**DOI:** 10.3389/fimmu.2026.1992961

**Published:** 2026-10-30

**Authors:** Jian-hao Zheng, Ruijie Ming, Yibo He, Xiao-xia Xi

**Affiliations:** 1 Department of Gastroenterology, Ningbo No.2 Hospital, Ningbo, China; 2 Department of Oncology, Chongqing University Three Gorges Hospital, Chongqing, China; 3 School of Medicine, Chongqing University, Chongqing, China; 4 Department of Clinical Laboratory, The First Affiliated Hospital of Zhejiang Chinese Medical University, Hangzhou, China

**Keywords:** colorectal cancer, deslanoside, GDI1, lymph node metastasis, molecular subtype

## Abstract

**Background:**

Lymph node metastasis (LNM) is central to colorectal cancer staging and treatment decisions and marks a critical transition from local growth to regional dissemination. The molecular heterogeneity associated with lymph node metastasis and the corresponding intervention leads require systematic investigation.

**Methods:**

This study established the C1 to C4 molecular subtypes from lymph node metastasis-related expression features. Multiple transcriptomic cohorts, clinical composition, pathway features, and TIDE, IPS, and ESTIMATE scores were integrated to characterize subtype differences. Analyses across multiple cohorts were used to screen candidate genes associated with progression. Cellular and subcutaneous xenograft experiments were performed to evaluate GDI1 function. The GDI1 structure was used for virtual screening and molecular dynamics analysis, followed by phenotypic evaluation of Deslanoside in cellular and xenograft models.

**Results:**

Four molecular subtypes displayed distinct expression profiles, clinical composition, and tumor microenvironment features. C4 showed coordinated enrichment of invasive, mesenchymal, hypoxia-related, and stromal programs together with algorithmic features associated with restricted immune effector activity. Analyses across multiple cohorts identified GDI1 as a candidate gene associated with adverse outcomes and progression. Two independent shRNAs reduced GDI1 expression, cell growth, and migration-related phenotypes in two colorectal cancer cell lines. GDI1 knockdown using shGDI1 #1 also reduced subcutaneous xenograft burden. Deslanoside ranked among the leading candidates in the computational screen. Deslanoside treatment produced antitumor phenotypes in cellular and xenograft models that were directionally concordant with those produced by GDI1 depletion.

**Conclusion:**

This study delineates the molecular heterogeneity associated with lymph node metastasis in colorectal cancer, supports a tumor-promoting role for GDI1, and provides a basis for further investigation of Deslanoside as a candidate compound.

## Introduction

Colorectal cancer continues to account for a substantial share of global cancer incidence and mortality (1). Lymph node status is central to disease staging and treatment decisions and also reflects the transition from local growth to regional dissemination (2). This process is jointly shaped by tumor cell states, stromal remodeling, lymphatic routes, and immune ecology. A single marker generally captures only one dimension of this process and cannot adequately represent differences in metastatic capacity and microenvironmental dependence among tumors. Defining molecular subtypes from lymph node metastasis-related expression features may therefore help identify progression-associated disease states within a unified framework and provide a clearer biological context for subsequent functional screening.

Studies of the colorectal cancer consensus molecular subtypes (CMS) have revealed systematic differences in epithelial programs, metabolic states, immune responses, and stromal infiltration and have defined a distinct type characterized by transforming growth factor-β (TGFβ) signaling and matrix remodeling in a stroma-rich setting (3). Multiregional transcriptomics further showed that intratumoral transcriptional heterogeneity and microenvironmental composition jointly influence CMS assignment, with stromal components making an important contribution to CMS4 classification (4). Integrated single-cell and bulk transcriptomic studies have further distinguished tumor epithelial cell-intrinsic states from contributions of the fibrotic microenvironment and have shown that colorectal cancer cell states and microenvironmental composition vary along a continuum (5, 6). Recent work in advanced colorectal cancer liver metastasis models and patient samples also showed that TGFβ can simultaneously restrict T cell recruitment and induce immunosuppressive SPP1+ macrophage states, thereby linking fibrotic stroma to immune escape (7). These findings indicate that molecular subtyping focused on lymph node metastasis should jointly resolve tumor intrinsic programs, stromal states, and the immune environment while interpreting stromal signals in bulk expression data within the context of tissue composition.

Moving from disease states to molecular candidates for intervention requires cross-cohort associations to be connected with functional perturbation. Rab-dependent vesicular trafficking regulates receptor recycling, membrane protein localization, and secretion, and its dysregulation can contribute to tumor cell migration and invasion (8, 9). GDP dissociation inhibitor 1 (GDI1) is an important regulator of the Rab cycle. Previous colorectal cancer cohort studies associated its high expression with aggressive clinical features and adverse outcomes (10). A recent siRNA study in HCT116 and HT29 cells also found reduced proliferation and migration accompanied by increased apoptosis after GDI1 silencing, providing independent *in vitro* evidence for a tumor-promoting role (11). Genetic studies in model organisms further showed that Rab GDI participates in invadopodia-related membrane trafficking and offers a mechanistic entry point for understanding its pro-invasive activity (12). Building on this evidence, the present study established colorectal cancer molecular subtypes from lymph node metastasis-related expression features, screened candidate genes across multiple cohorts, and evaluated GDI1 function using two independent short hairpin RNAs (shRNAs) in HCT116 and SW480 cells before extending the validation to subcutaneous xenografts. The GDI1 structure was subsequently used for virtual screening and molecular dynamics analysis, followed by phenotypic evaluation of Deslanoside in cellular and xenograft models. This design connected molecular subtype identification, functional candidate validation, and formulation of a pharmacologic hypothesis that remains to be tested. Rather than treating GDI1 as an unstudied target, the study extended prior evidence by adding independent perturbation in SW480 cells, subcutaneous xenograft validation, and a structure-guided candidate-compound analysis.

## Results

### Lymph node metastasis-related expression features define molecular subtypes of colorectal cancer

To resolve the molecular heterogeneity associated with lymph node metastasis in colorectal cancer, we first evaluated the survival associations of lymph node metastasis-related genes. The forest plot linked GABRD, MPP2, PIANP, GDI1, and ATP6V1B1 to higher risk, whereas OXSM, NUDT6, LARS2, PSMA5, and GSR showed associations in the opposite direction, with GDI1 ranking among the leading risk-associated genes (**Figure 1A**). These outcome-associated candidates formed the expression feature set used for subsequent molecular subtyping. Consensus clustering based on this feature set showed that all clusters retained high internal consistency under the four-cluster solution when cluster-consensus scores were compared across candidate cluster numbers. The corresponding consensus matrix formed clearly delineated blocks, supporting the classification of samples into four molecular subtypes designated C1, C2, C3, and C4 (**Figures 1B, C**).

**Figure 1:**
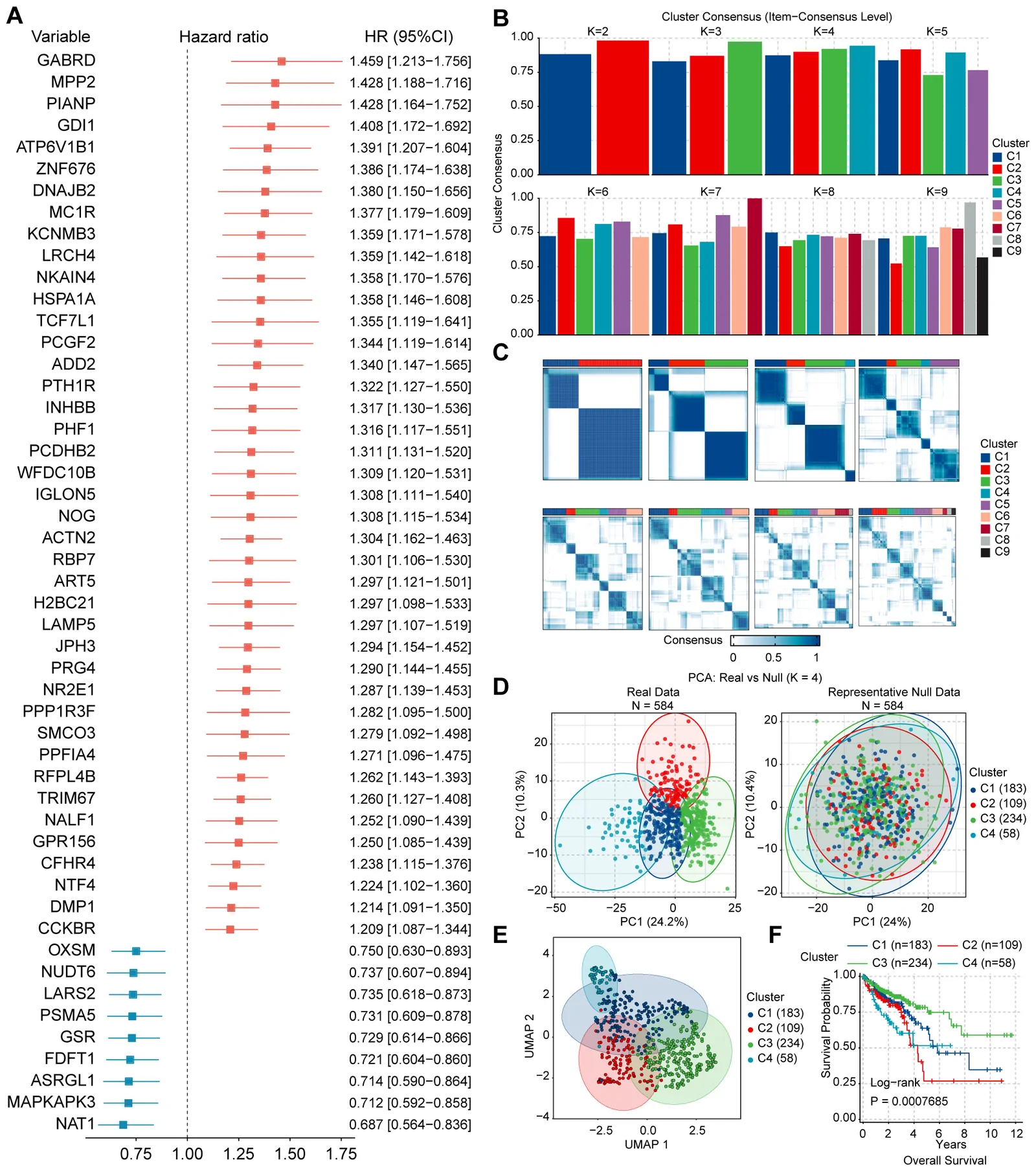
Molecular subtyping based on lymph node metastasis-related expression features in colorectal cancer. **(A)** Univariable Cox regression forest plot showing hazard ratios and confidence intervals for candidate genes. **(B)** Cluster-consensus scores across candidate solutions from K = 2 to K = 9, calculated as the mean pairwise consensus among samples within each cluster. **(C)** Consensus matrices across different K values. **(D)** Principal component analysis of the observed data and representative null-model data at K = 4. **(E)** UMAP distribution of the four molecular subtypes. **(F)** Kaplan-Meier overall survival curves for the four molecular subtypes.

Principal component analysis further showed that the four molecular subtypes in the observed data formed a clustering structure distinct from that of the representative null dataset (**Figure 1D**). Uniform manifold approximation and projection (UMAP) provided a complementary view of subtype distribution, with the subtypes occupying relatively distinct expression spaces while retaining a degree of continuity between them (**Figure 1E**). The four molecular subtypes also displayed different overall survival curves in the discovery cohort (**Figure 1F**).

### Molecular subtypes exhibit distinct expression profiles and clinical compositions

Each subtype displayed a distinct differential expression profile relative to the remaining samples. C1 was characterized by upregulation of SPOCK1, TNS1, FLT1, HIP1, and KCNE4, which are involved in extracellular matrix, cell adhesion, and vascular processes. Representative upregulated genes in C2 included INAFM1, ZNF316, MOSPD3, and YPEL3, revealing a transcriptional regulatory profile distinct from those of the other subtypes. C3 was marked by upregulation of PPA2, COX5A, NAT1, UQCRFS1, and GSR, primarily pointing to mitochondrial metabolism and redox processes. C4 showed prominent expression of ZFHX4, FAM43B, NRK, PNMA8B, and NALCN, forming a readily distinguishable combination of subtype markers (**Figure 2A**). When the marker gene templates for the four subtypes were applied to The Cancer Genome Atlas colorectal cancer (TCGA-CRC) cohort, GSE12945, GSE13294, GSE14333, GSE17536, GSE39582, GSE41258, and other Gene Expression Omnibus (GEO) cohorts, expression patterns corresponding to C1 through C4 were identified in each dataset, indicating that the principal expression features of the four subtypes were consistent across cohorts (**Figure 2B**).

**Figure 2:**
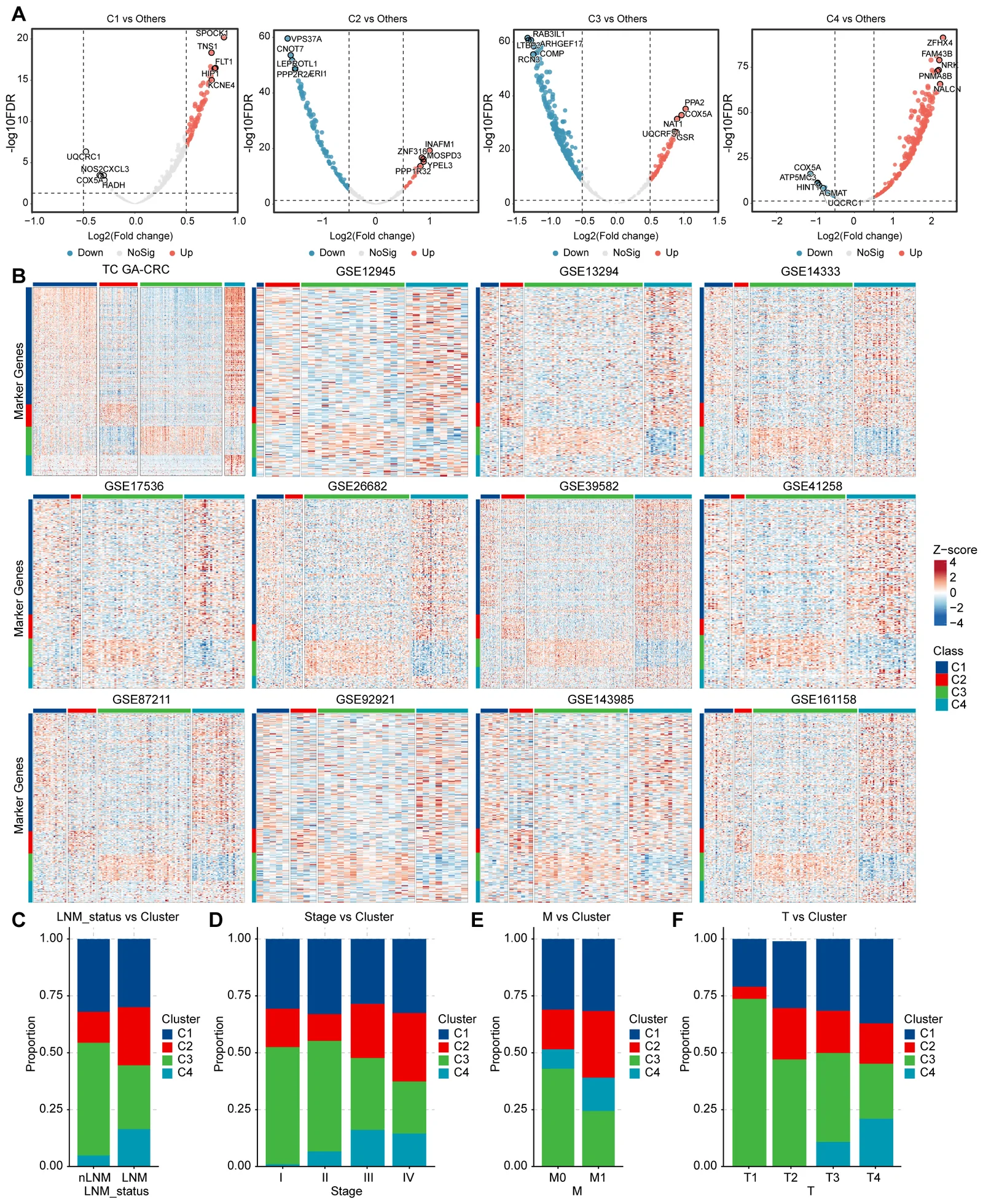
Expression characteristics and clinical composition of the four molecular subtypes. **(A)** Differential expression volcano plots comparing C1, C2, C3, and C4 separately with all remaining samples. Colors distinguish genes that are upregulated, downregulated, or do not reach the threshold. **(B)** Heatmaps of standardized expression for molecular subtype marker genes and template assignments across TCGA-CRC and multiple GEO cohorts. **(C)** Subtype composition in nLNM and LNM samples. **(D)** Subtype composition across clinical stages. **(E)** Subtype composition in M0 and M1 samples. **(F)** Subtype composition across T stages. LNM, lymph node metastasis; nLNM, non-lymph-node-metastasis. Panels **(C–F)** were evaluated using global Pearson chi-square tests. P values were 3.53 × 10⁻^10^, 1.30 × 10⁻^4^, 0.00339, and 0.000136, respectively. P values are unadjusted and describe exploratory associations in the discovery cohort.

Subtype composition varied according to lymph node metastasis (LNM) status. Compared with non-lymph-node-metastasis (nLNM) samples, LNM samples contained higher proportions of C2 and C4 and a lower proportion of C3 (**Figure 2C**). Across clinical stages, samples from later stages showed greater representation of C2 and C4 together with reduced representation of C3 (**Figure 2D**). M1 samples similarly showed increases in C2 and C4 and a decrease in C3 (**Figure 2E**). As the extent of local invasion by the primary tumor increased, the proportion of C4 rose further at higher T stages (**Figure 2F**). These compositional changes linked C2 and C4 to lymph node metastasis, distant metastasis, and clinical progression, with the most concentrated changes observed in C4. Global Pearson chi-square tests showed associations of subtype composition with LNM status (n = 581, P = 3.53 × 10⁻^10^), clinical stage (n = 564, P = 1.30 × 10⁻^4^), M stage (n = 517, P = 0.00339), and T stage (n = 582, P = 0.000136). These are exploratory associations in the discovery cohort; the LNM comparison is descriptive because LNM status was used to select the clustering features. Microsatellite instability (MSI) status was recorded for 366 of 584 samples, comprising 54 MSI-high (MSI-H) and 312 microsatellite-stable (MSS) samples; 218 samples had missing MSI annotations. Among samples with known MSI status, MSI-H was present in 18 of 139 C1 samples (12.9%), 1 of 47 C2 samples (2.1%), 29 of 130 C3 samples (22.3%), and 6 of 50 C4 samples (12.0%).

### C4 is enriched for invasive, stromal, and hypoxia-related molecular programs

Hallmark gene set analysis showed that C4 was enriched for programs related to epithelial-mesenchymal transition (EMT), hypoxia, Notch signaling, Wnt/β-catenin signaling, KRAS signaling, myogenesis, complement, and apical junctions. C3 was primarily characterized by oxidative phosphorylation, cell cycle, MYC target, DNA repair, fatty acid metabolism, and p53 pathway programs (**Figure 3A**). Kyoto Encyclopedia of Genes and Genomes (KEGG) pathway analysis also highlighted matrix-associated programs in C4. C4 showed higher scores for focal adhesion, PI3K-Akt signaling, Rap1 signaling, extracellular matrix receptor interaction, and cell adhesion molecule pathways, whereas C3 was distinguished by enrichment of oxidative phosphorylation and multiple metabolism-related pathways (**Figure 3B**). PROGENy analysis further separated the signaling activities of the subtypes. TGFβ and hypoxia signaling were prominent in C4, whereas C3 showed higher PI3K, WNT, MAPK, NF-κB, TNFα, and VEGF signaling activities (**Figure 3C**). CancerSEA functional state scores were generally higher in C4 for epithelial-mesenchymal transition, invasion, metastasis, angiogenesis, inflammation, and hypoxia, whereas C3 showed greater activity in cell cycle and DNA repair programs (**Figure 3D**). Mean Hallmark WNT/β-catenin scores for C1, C2, C3, and C4 were 0.153, 0.068, −0.121, and 0.240, respectively; mean KEGG PI3K-Akt scores were 0.081, 0.043, 0.012, and 0.138. In contrast, mean PROGENy WNT scores were −0.392, −0.141, 0.739, and −1.478, and mean PROGENy PI3K scores were −0.205, −0.113, 0.440, and −0.917. Hallmark and KEGG ssGSEA scores summarize expression enrichment within annotated gene sets, whereas PROGENy infers activity from weighted downstream transcriptional responses to pathway perturbation. These scores therefore measure different features and should be compared within, rather than across, scoring methods.

**Figure 3:**
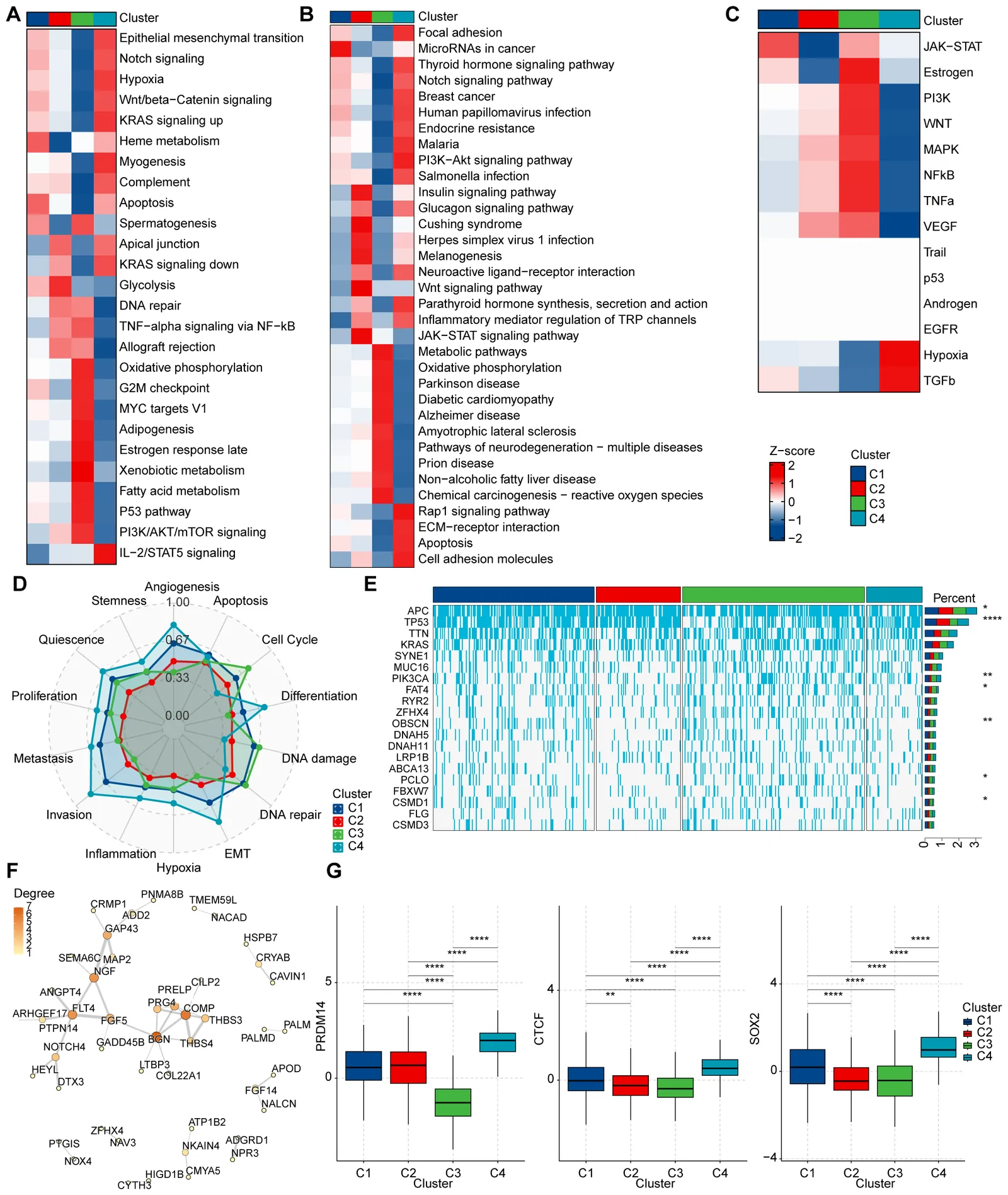
Functional pathways, somatic mutations, and transcriptional regulatory features of the four molecular subtypes. **(A)** Heatmap of Hallmark gene set scores. **(B)** Heatmap of KEGG pathway scores. **(C)** Heatmap of PROGENy pathway activity. **(D)** Radar plot of CancerSEA functional state scores. **(E)** Somatic mutation matrix arranged by subtype and comparison of mutation frequencies. **(F)** Protein interaction network of subtype-associated genes, with node color indicating connectivity. **(G)** Distribution of PRDM14, CTCF, and SOX2 transcription factor activity across the four molecular subtypes. * denotes P < 0.05, ** denotes P < 0.01, *** denotes P < 0.001, and **** denotes P < 0.0001.

The somatic mutation landscape revealed different subtype distributions of frequently mutated genes including APC, TP53, KRAS, PIK3CA, FAT4, and ZFHX4, with TP53 mutations being more concentrated in C2 (**Figure 3E**). Subtype-associated genes formed several protein interaction modules that included FLT4, NGF, COMP, THBS3, THBS4, PRELP, PRG4, and BGN as nodes related to extracellular matrix organization and tissue remodeling (**Figure 3F**). Transcription factor activity analysis showed higher activities of PRDM14, CTCF, and SOX2 in C4 (**Figure 3G**). The combined pathway, functional state, mutation, interaction network, and transcription factor activity features further defined the invasive and stromal molecular profile of C4.

### C4 exhibits an immunosuppressive profile and low predicted responsiveness to immunotherapy

Tumor Immune Dysfunction and Exclusion (TIDE) analysis showed marked differences in predicted immunotherapy response across the molecular subtypes. Predicted responders constituted a higher proportion of C3, whereas predicted nonresponders predominated in C4 (**Figure 4A**). Consistent with this distribution, TIDE, Dysfunction, Exclusion, and cancer-associated fibroblast (CAF) scores were generally higher in C4 and lower in C3 (**Figure 4B**). Immunophenoscore (IPS) analysis revealed subtype differences across a complementary set of immune-related measures. The four weighted components—major histocompatibility complex (MHC), effector cells (EC), suppressor cells (SC), and checkpoints/immunomodulators (CP)—and their aggregate score (AZ) varied across subtypes, with a lower composite IPS in C4 and a higher composite IPS in C3 (**Figure 4C**).

**Figure 4:**
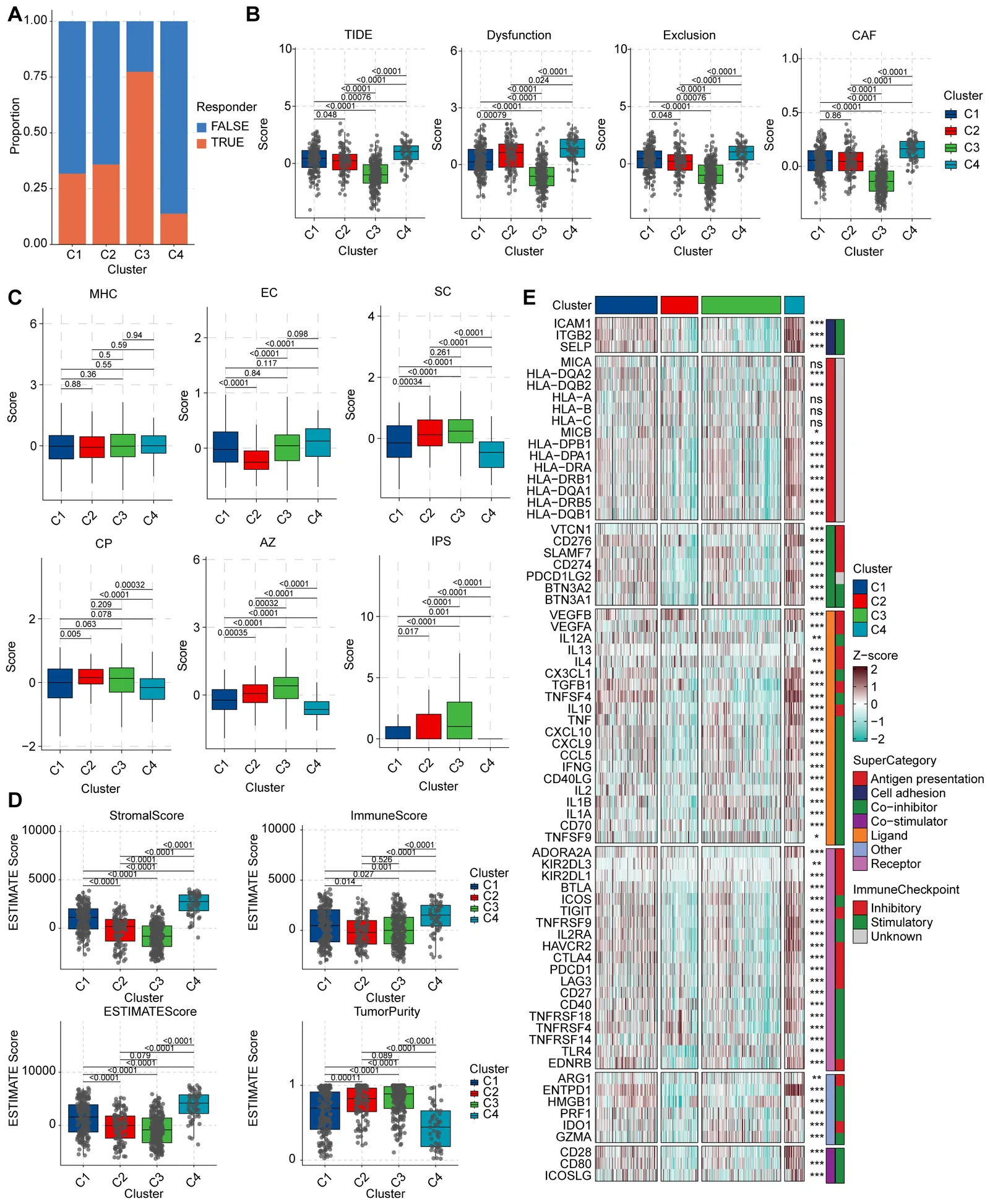
Immune microenvironment and predicted immunotherapy response across the four molecular subtypes. **(A)** Composition of immunotherapy responders and nonresponders predicted using TIDE. **(B)** Distribution of TIDE, Dysfunction, Exclusion, and CAF scores. **(C)** Distribution of the four weighted IPS components, their aggregate score, and the composite IPS. MHC, major histocompatibility complex and antigen-processing components; EC, effector cells; SC, suppressor cells; CP, checkpoints and immunomodulators; AZ, aggregate weighted z-score calculated as MHC + CP + EC + SC; IPS, immunophenoscore. **(D)** Distribution of StromalScore, ImmuneScore, ESTIMATEScore, and TumorPurity. **(E)** Heatmap of sample-level standardized expression of immunoregulatory genes annotated by antigen presentation, cell adhesion, co-inhibition, co-stimulation, ligand, receptor, and other categories.

ESTIMATE analysis showed that C4 had higher stromal, immune, and composite scores together with lower tumor purity. C3 contained a comparatively smaller stromal component (**Figure 4D**). The expression profiles of immunoregulatory molecules further defined the immune signaling composition of each subtype. Antigen presentation molecules including HLA-DPA1, HLA-DPB1, and HLA-DRA and coinhibitory checkpoints including TIGIT, CTLA4, PDCD1, LAG3, and HAVCR2 showed higher expression in C4. Costimulatory receptors including ICOS, TNFRSF9, TNFRSF18, TNFRSF4, and TNFRSF14 and cytokines including TGFB1, IL10, CXCL9, CXCL10, and CCL5 also showed coordinated changes (**Figure 4E**). Together, these features characterized C4 as a microenvironmental state with enriched stromal and immune components, heightened immunoregulatory signaling, and low predicted responsiveness to immunotherapy.

### Multi-cohort screening identifies GDI1 as a candidate gene associated with adverse prognosis

To identify core molecules associated with colorectal cancer progression, we conducted a multi-cohort survival association analysis of the candidate genes. The cross-cohort risk map showed a consistent risk direction for GDI1 in GSE17536, GSE39582, GSE41258, GSE71187, and TCGA-CRC, with GDI1 ranking first by mean risk. RTL8C, HSPB1, CDIPT, THBS3, AIP, and RCN3 followed in the ranking (**Figure 5A**). Although RTL8C and HSPB1 also showed hazard ratios above 1, their mean hazard ratios were lower and statistical significance was observed in only two of five cohorts, whereas GDI1 showed a hazard ratio above 1 and statistical significance in all five cohorts. GDI1 also ranked among the leading genes in the preceding survival analysis of lymph node metastasis-related genes, and the convergence of the two screening approaches prioritized GDI1 as the lead candidate for subsequent investigation. Kaplan-Meier analysis further showed poorer overall survival outcomes in the GDI1 high-expression group across TCGA-CRC and multiple GEO cohorts (**Figure 5B**). The high-expression group also showed consistently adverse directions for disease-specific survival, disease-free survival, and progression-free survival in TCGA-CRC. The association between GDI1 and patient outcome therefore extended across cohorts and survival endpoints.

**Figure 5:**
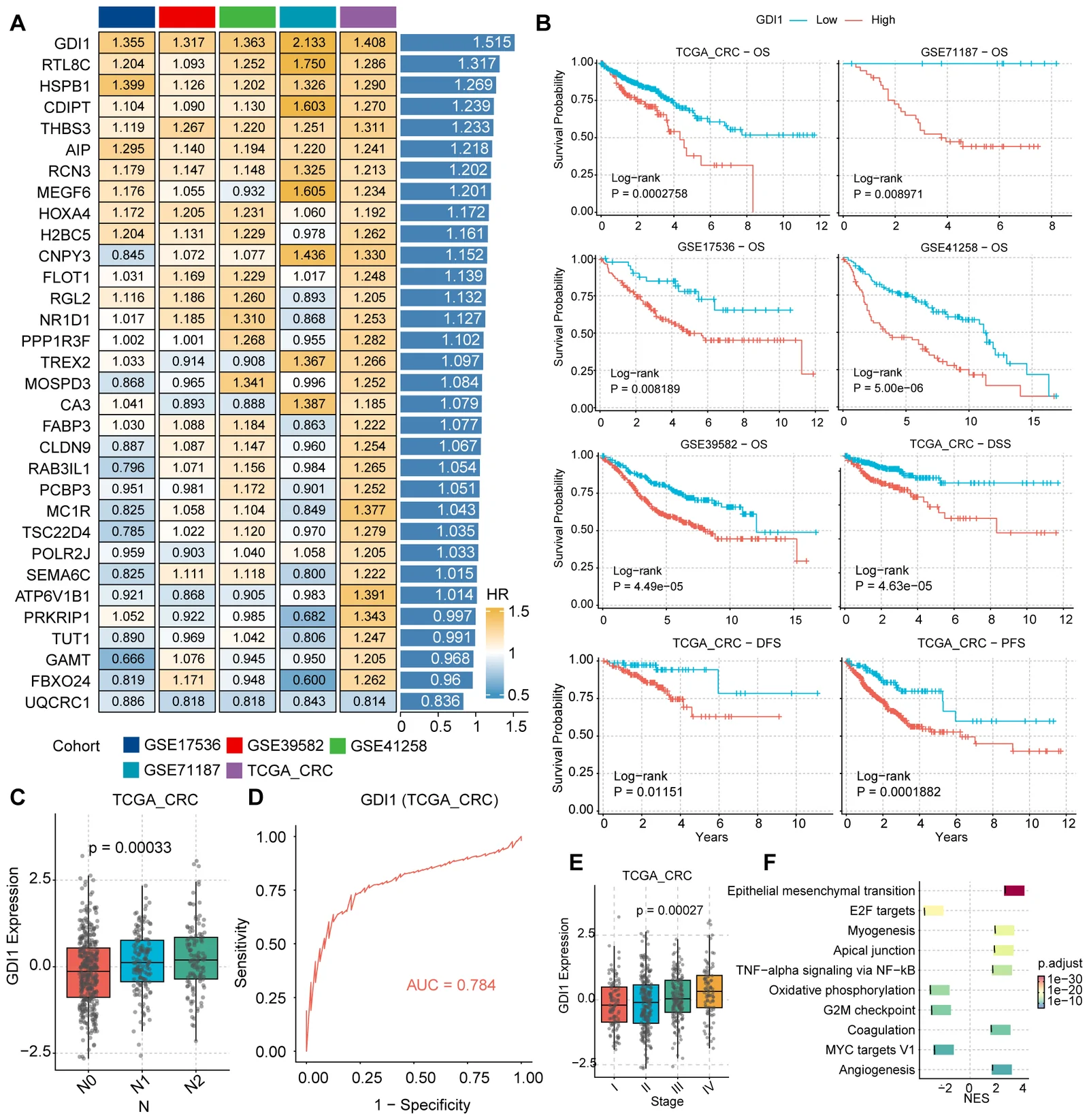
Multi-cohort identification of GDI1 and its associations with clinical features. **(A)** Heatmap of univariable Cox regression results for candidate genes in GSE17536, GSE39582, GSE41258, GSE71187, and TCGA-CRC. The mean cross-cohort hazard ratio is shown on the right. **(B)** Kaplan-Meier curves for the GDI1 high-expression and low-expression groups across different cohorts and survival endpoints. **(C)** GDI1 expression across N stages. **(D)** Receiver operating characteristic curve for distinguishing lymph node metastasis status using GDI1. **(E)** GDI1 expression across clinical stages. **(F)** Gene set enrichment analysis results for the GDI1 high-expression and low-expression groups.

At the clinical phenotype level, GDI1 expression increased with advancing lymph node stage (**Figure 5C**), and its expression distinguished LNM from nLNM status (**Figure 5D**). The same direction was observed across clinical stages, with higher GDI1 expression in samples from later stages (**Figure 5E**). Gene set enrichment analysis associated high GDI1 expression with epithelial-mesenchymal transition, myogenesis, apical junction, TNFα/NF-κB signaling, coagulation, and angiogenesis, whereas oxidative phosphorylation, cell cycle, and MYC target programs showed the opposite direction (**Figure 5F**). These findings closely linked GDI1 to colorectal cancer progression, adverse survival, and invasion-related transcriptional states.

### GDI1 knockdown suppresses colorectal cancer cell growth and migration and reduces subcutaneous xenograft growth

To examine the function of GDI1, we introduced two independent shRNAs into HCT116 and SW480 cells. Reverse transcription quantitative polymerase chain reaction (RT-qPCR) showed that shGDI1 #1 and shGDI1 #2 both effectively reduced GDI1 transcript levels in the two cell lines, with consistent effects across the two shRNA sequences (**Figure 6A**). CCK-8 assays showed lower growth curves in both HCT116 and SW480 cells after GDI1 knockdown, with the differences widening over time in culture (**Figure 6B**). Representative Transwell images showed markedly fewer cells crossing the membrane with either shRNA, and quantification showed the same direction in both cell lines (**Figures 6C, D**). Wound healing assays likewise showed that GDI1 knockdown groups retained wider wound areas during the observation period and had lower closure rates than the NC groups (**Figures 6E, F**).

**Figure 6:**
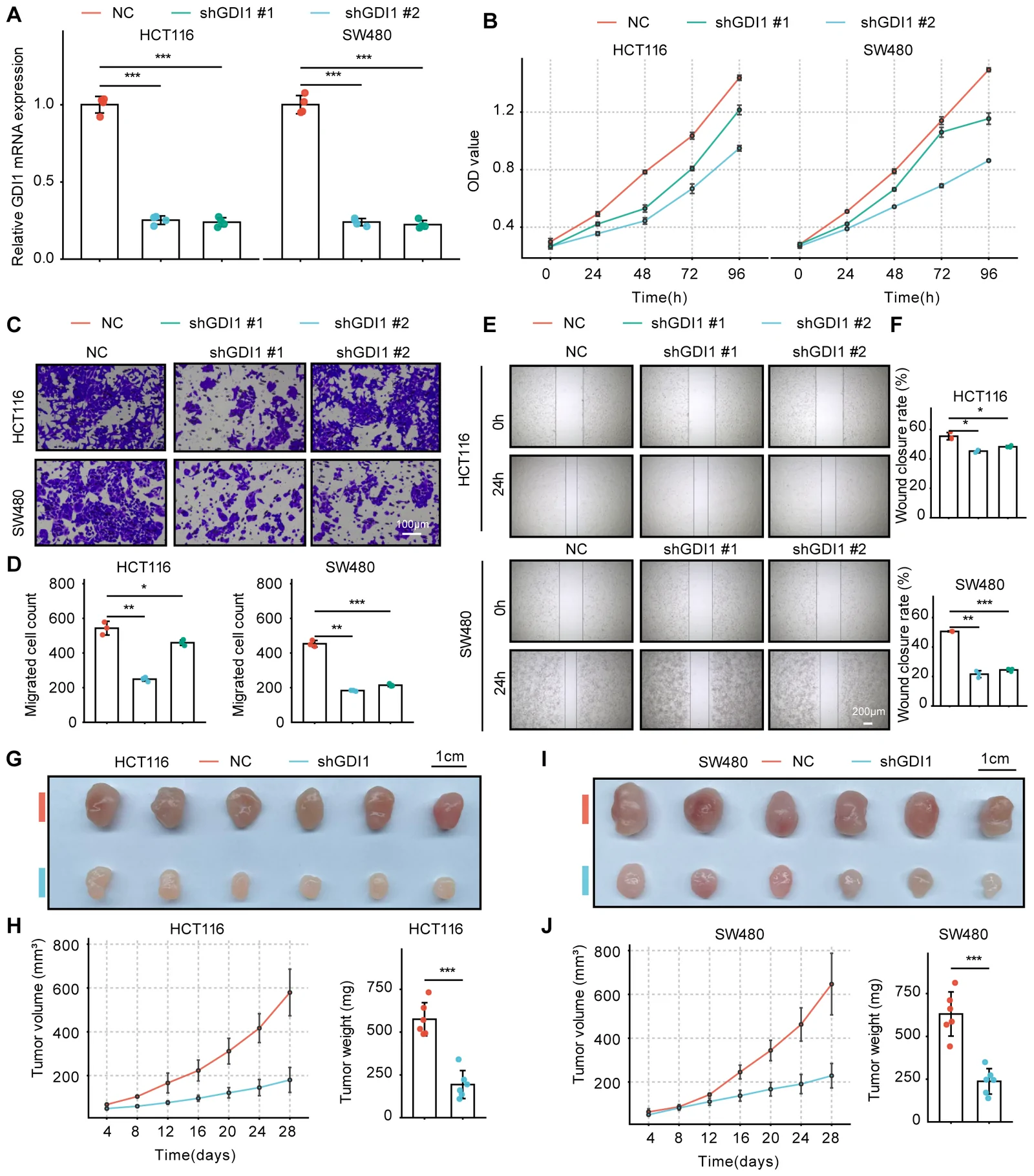
GDI1 knockdown suppresses colorectal cancer cell growth, migration, and subcutaneous xenograft growth. **(A)** Relative GDI1 mRNA expression in HCT116 and SW480 cells from the NC, shGDI1 #1, and shGDI1 #2 groups. **(B)** CCK-8 assay results in HCT116 and SW480 cells. **(C, D)** Representative Transwell migration images and migrated cell counts for HCT116 and SW480 cells after 24 h of incubation following seeding. Scale bar, 100 μm. **(E, F)** Representative wound-healing images and wound closure measurements for HCT116 and SW480 cells. Scale bar, 200 μm. **(G, H)** Xenograft images, tumor volume curves, and endpoint tumor weights for HCT116 cells in the NC and shGDI1 #1 groups. Scale bar, 1 cm. **(I, J)** Xenograft images, tumor volume curves, and endpoint tumor weights for SW480 cells in the NC and shGDI1 #1 groups. Scale bar, 1 cm. * denotes P < 0.05, ** denotes P < 0.01, and *** denotes P < 0.001.

In the HCT116 subcutaneous xenograft setting, the shGDI1 #1 group formed smaller endpoint tumors, maintained a lower tumor volume curve than the NC group, and showed a corresponding reduction in endpoint tumor weight (**Figures 6G, H**). SW480 xenografts yielded findings in the same direction, with the shGDI1 #1 group showing lower tumor burden in gross tumor appearance, growth curves, and endpoint tumor weight (**Figures 6I, J**). The concordant *in vitro* findings from both cell lines and their corresponding *in vivo* tumorigenicity findings indicate that GDI1 promotes colorectal cancer cell growth and migration as well as xenograft growth.

### Virtual screening and molecular dynamics simulations identify Deslanoside as a candidate GDI1 ligand

Structure-based virtual screening against the three-dimensional structure of GDI1 yielded a group of compounds with favorable predicted docking scores. Among 2, 990 compounds with successful docking results, Deslanoside and Amphotericin B shared the most favorable predicted score of −10.40 kcal/mol, followed by Suramin (−10.37 kcal/mol), Zotarolimus (−10.26 kcal/mol), Rutin (−10.25 kcal/mol), and Ledipasvir (−10.23 kcal/mol) among the leading candidates (**Figure 7A**). Surface and Cartoon representations of the predicted Deslanoside-GDI1 complex placed Deslanoside within a predicted binding pocket on the GDI1 surface (**Figure 7B**). An enlarged view of the pocket showed predicted contacts between Deslanoside and residues including GLU233, GLN236, SER45, TYR38, ARG35, and ARG55. These residues surrounded the compound in the predicted pose.

**Figure 7:**
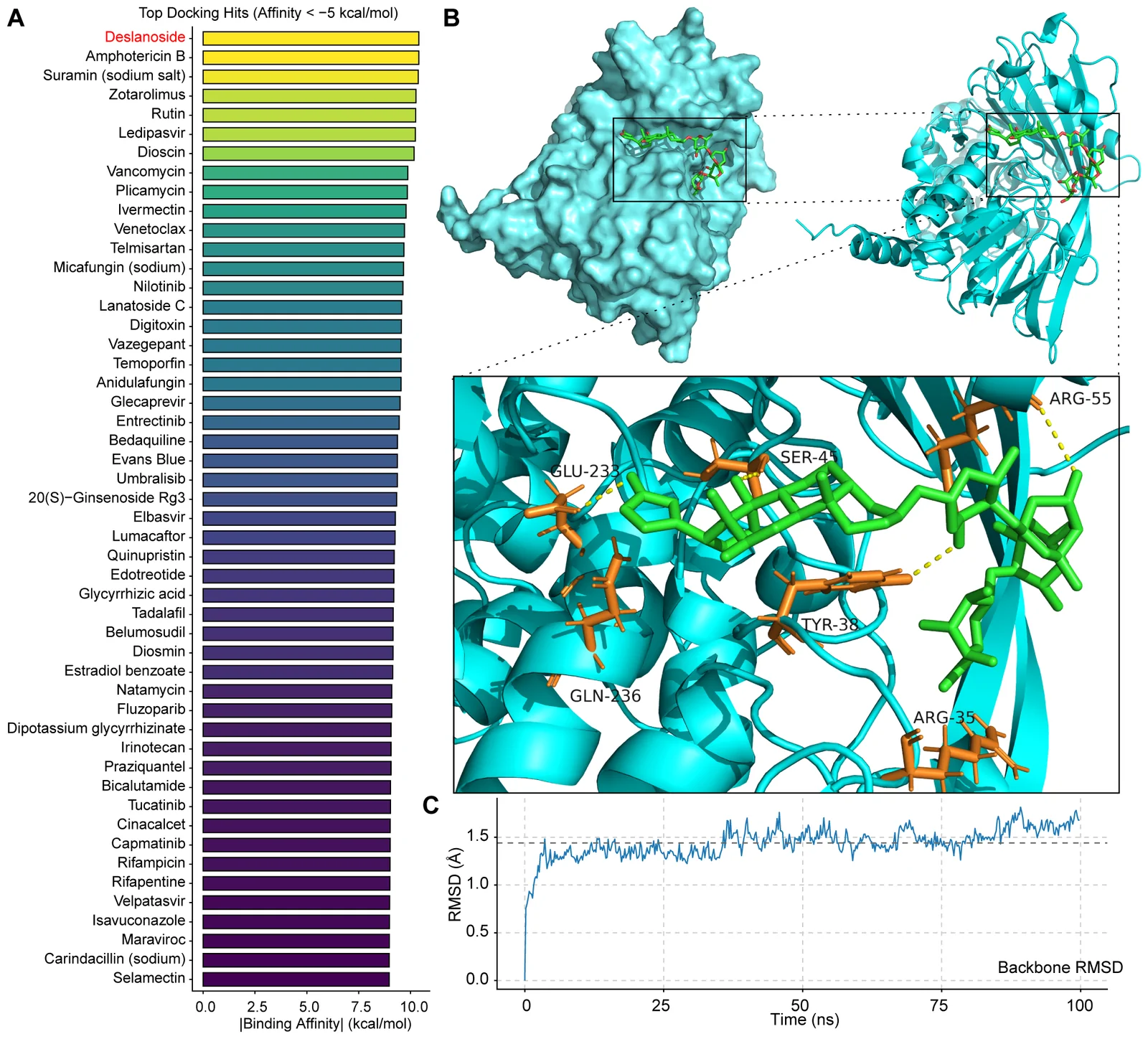
Virtual screening, predicted binding conformation, and molecular dynamics simulation of candidate compounds associated with GDI1. **(A)** Leading candidate compounds identified by virtual screening against GDI1, with Deslanoside among the top-ranked candidates. **(B)** Surface structure, cartoon structure, and enlarged binding pocket view of the predicted Deslanoside and GDI1 complex, with key residues involved in the predicted contacts labeled. **(C)** Protein backbone root mean square deviation of the predicted Deslanoside and GDI1 complex during the 100 ns molecular dynamics simulation.

Molecular dynamics simulations showed a rapid initial adjustment in the backbone root mean square deviation of the Deslanoside-GDI1 complex, followed by a relatively stable range of fluctuation that persisted throughout the later trajectory (**Figure 7C**). The virtual screening, binding pose, and dynamic simulation results prioritized Deslanoside for downstream phenotypic evaluation based on candidate ranking, spatial fit, and dynamic behavior; they did not establish direct binding to GDI1.

### Deslanoside and GDI1 knockdown produce consistent antitumor phenotypes

To examine the functional relationship between Deslanoside and GDI1, we established four groups in HCT116 and SW480 cells consisting of NC, NC plus Deslanoside, shGDI1 #1 plus DMSO, and shGDI1 #1 plus Deslanoside. CCK-8 assays showed that Deslanoside exposure and GDI1 knockdown each lowered the growth curves of both cell lines, while the combination groups likewise remained at low levels (**Figure 8A**). Transwell assays showed fewer migrating cells with either Deslanoside exposure or GDI1 knockdown than in the NC groups. Adding Deslanoside after GDI1 knockdown produced migrating cell numbers similar to those observed with GDI1 knockdown alone (**Figures 8B, C**). Wound healing assays yielded concordant results, with both Deslanoside exposure and GDI1 knockdown reducing wound closure rates and the combination groups showing closure phenotypes similar to those of the GDI1 knockdown groups (**Figures 8D, E**).

**Figure 8:**
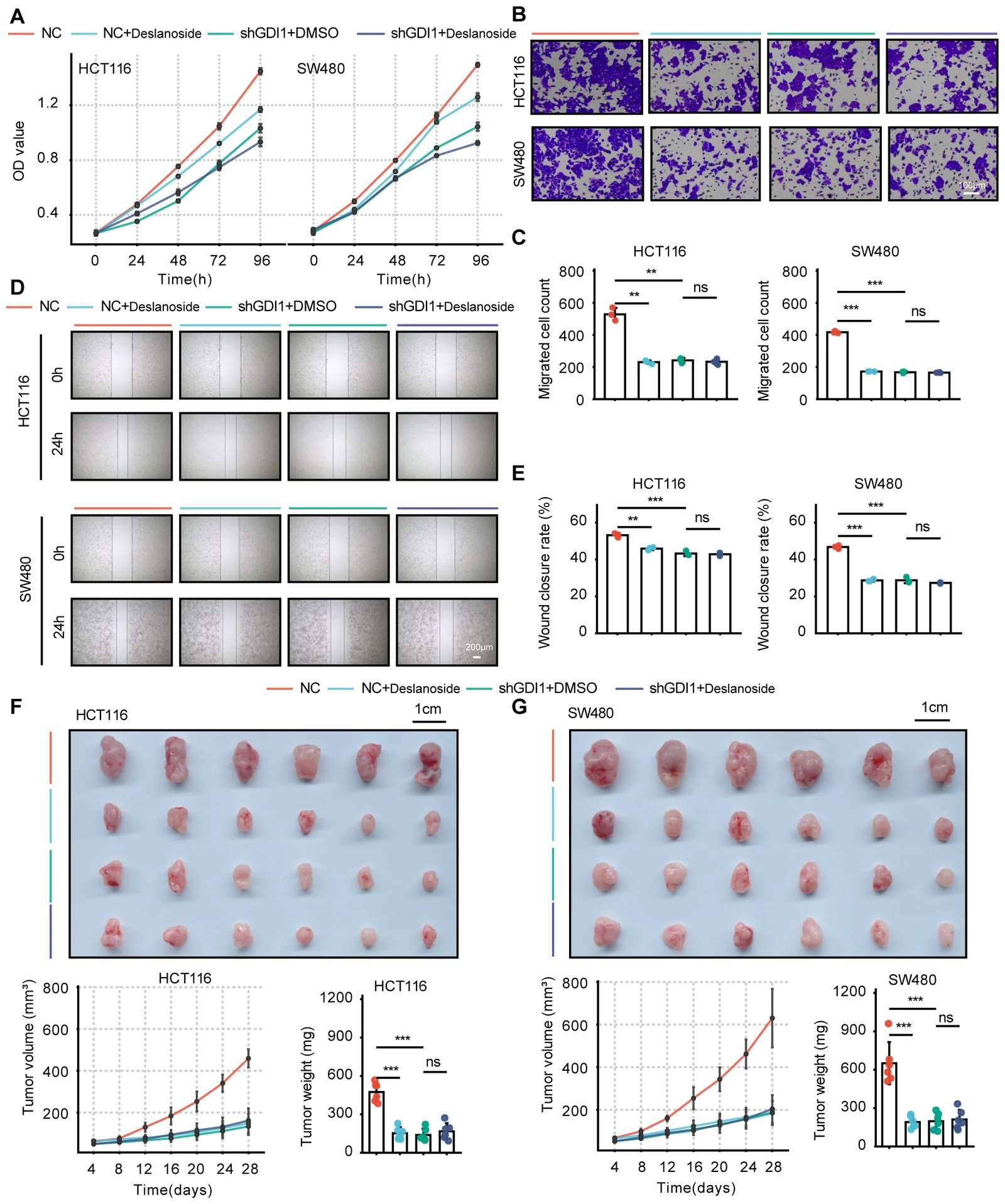
Combined effects of Deslanoside and GDI1 knockdown on malignant phenotypes in colorectal cancer. Experiments included the NC, NC plus Deslanoside, shGDI1 #1 plus DMSO, and shGDI1 #1 plus Deslanoside groups. **(A)** CCK-8 assay results in HCT116 and SW480 cells measured at 0, 24, 48, 72, and 96 h after treatment initiation. Cells were cultured overnight before treatment, which was maintained until each measurement. **(B, C)** Representative Transwell migration images and migrated cell counts for both cell lines pretreated for 24 h before seeding into the inserts and then incubated for 24 h. Scale bar, 100 μm. **(D, E)** Representative wound-healing images acquired at 0 and 24 h and wound closure measurements for both cell lines. Wounds were made 12 h after seeding, followed by treatment for 24 h. Scale bar, 200 μm. **(F)** Xenograft images, tumor volume curves, and endpoint tumor weights for the four HCT116 groups. Scale bar, 1 cm. **(G)** Xenograft images, tumor volume curves, and endpoint tumor weights for the four SW480 groups. Scale bar, 1 cm. ** denotes P < 0.01, *** denotes P < 0.001, and ns denotes not significant (P ≥ 0.05).

In HCT116 subcutaneous xenografts, Deslanoside administration and GDI1 knockdown each reduced the tumor growth curve and endpoint tumor weight, while the endpoint tumor weight of the combination group was similar to that of the GDI1 knockdown group (**Figure 8F**). SW480 xenografts reproduced the same direction of change (**Figure 8G**). The consistent findings across *in vitro* cell growth, Transwell migration, wound closure, and both xenograft settings were directionally concordant with the GDI1 knockdown phenotypes and therefore support further testing of a pharmacological relationship between Deslanoside and GDI1; they do not establish direct target engagement or causal epistasis.

## Discussion

This study established an integrated evidence chain centered on heterogeneity associated with lymph node metastasis. Expression features associated with lymph node metastasis stratified colorectal cancer into molecular subtypes with distinct clinical composition, molecular programs, and immune microenvironments. Analyses across multiple cohorts and perturbation experiments *in vitro* and *in vivo* supported GDI1 as a tumor-promoting candidate. Computational structural analyses and pharmacologic experiments provided the basis for subsequent mechanistic investigation of Deslanoside. The molecular subtype analysis defined progression-associated disease states, GDI1 served as the molecular entry point for functional perturbation, and Deslanoside offered a testable intervention lead. This sequence advances expression associations toward functional validation and a pharmacologic hypothesis.

C4 was most prominently characterized by coordinated activation of invasive, mesenchymal, hypoxia-related, and matrix-related programs and was enriched among samples with lymph node metastasis and more advanced clinical characteristics. The canonical CMS classification likewise defines CMS4 by active TGFβ signaling and stromal infiltration (3). Multiregional transcriptomics further showed that CMS assignment is jointly influenced by intratumoral transcriptional heterogeneity and microenvironmental composition, with stromal components exerting a substantial effect on CMS4 classification (4). Single-cell studies have also shown that tumor epithelial cell-intrinsic states, the extent of fibrosis, and microenvironmental composition jointly shape bulk classifications (5, 6). C4 was derived from LNM- and survival-associated features, whereas CMS provides a broader colorectal cancer classification. Its resemblance to CMS4 refers to shared stromal and mesenchymal programs; it does not establish classification concordance or equivalence between C4 and CMS4. A recent study integrating single-cell and spatial transcriptomics linked EMT-active malignant epithelial cells and fibroblast states to colorectal cancer metastasis, further supporting an interpretation of invasive phenotypes that integrates cellular states with tissue ecology (13). The enrichment of a Hallmark Wnt/β-catenin gene set alongside ECM-receptor interaction and focal adhesion programs in C4 can be considered in the context of Wnt-related regulation of colorectal cancer cell fate, migration, and the microenvironment (14).

Another defining feature of C4 was the coexistence of stromal enrichment and restricted immune effector activity. This subtype had higher stromal and immune-related scores together with higher CAF and immune exclusion scores and coordinated changes across multiple immunoregulatory signals, indicating that enriched immune components coexisted with stromal constraint. Recent work on advanced colorectal cancer liver metastasis showed that TGFβ can simultaneously restrict T cell recruitment and induce immunosuppressive SPP1+ macrophage states, creating a composite barrier that connects fibrotic stroma with immune escape (7). Multi-omics research has also linked CAF features in colorectal cancer to EMT, angiogenesis, lymphatic invasion, and immunosuppressive states (15). Studies in immunocompetent mouse colorectal cancer models further showed that ZEB1-dependent CAF polarization linked collagen deposition, inflammatory signaling, lymphocyte recruitment, and sensitivity to immune checkpoint blockade (16). Together with the lower IPS and higher TIDE exclusion scores, these observations suggest that stromal enrichment in C4 may coexist with restricted antitumor immune activity, providing a rationale to investigate TGFβ- and CAF-associated mechanisms in this subtype. The WNT and PI3K results are method-dependent and do not establish C4 as uniformly WNT-high or PI3K-high. TIDE, IPS, and ESTIMATE are transcriptome-derived measures and cannot establish clinical benefit from immune checkpoint blockade. MSI annotations were incomplete and unevenly distributed across subtypes, and the clinical annotation used for this analysis did not include matched colon-versus-rectum site information. The immune comparisons were not adjusted for MSI or tumor site, so these factors may contribute to the observed differences.

GDI1 linked the progression-associated states represented by the molecular subtypes to reproducible functional phenotypes. Rab proteins regulate receptor localization, secretory activity, and structures involved in cell motility through membrane trafficking, and their dysregulation can promote diverse tumor phenotypes (8, 9). Previous colorectal cancer studies associated high GDI1 expression with aggressive features and adverse outcomes (10). A recent siRNA study in HCT116 and HT29 cells likewise found reduced proliferation and migration accompanied by increased apoptosis after GDI1 silencing (11). The current study found concordant associations across multiple cohorts, observed consistent growth and migration-related phenotypes with two independent shRNAs in HCT116 and SW480 cells, and subsequently observed lower tumor burden in two subcutaneous xenograft models. These findings extend prior cohort associations and *in vitro* functional evidence by adding replication with independent perturbations, an additional colorectal cancer cell line, and evidence from *in vivo* tumor growth. Work in model organisms implicating GDI1 in invadopodia-associated membrane trafficking provides a specific starting point for examining receptor recycling, membrane protein localization, and invasive structures in colorectal cancer cells (12).

Pharmacologic investigation further introduced Deslanoside as a candidate intervention. Previous work observed changes in growth-related and migration-related phenotypes after Deslanoside treatment in prostate cancer cells and xenograft models (17). The related cardiac glycoside Oleandrin also elicited antitumor phenotypes in colorectal cancer cells and patient-derived colon cancer organoids, with Na+/K+-ATPase and GRP78 stress regulation implicated in these effects (18). The canonical pharmacology of cardiac glycosides involves Na+/K+-ATPase inhibition and downstream signal regulation (19). Work in colon cancer has also linked Na+/K+-ATPase activity to Wnt signaling and macropinocytosis (20). The favorable docking rank and existing pharmacological and preclinical background support further investigation of Deslanoside without establishing GDI1 selectivity. In this study, docking and molecular dynamics provided a predicted interaction, while Deslanoside treatment and GDI1 depletion produced directionally concordant phenotypes. Direct binding and GDI1-dependent drug action remain unproven in the absence of target-engagement and rescue experiments. Cardiac glycoside pharmacology also raises a safety concern for systemic anticancer treatment. Our intratumoral xenograft experiments do not define tumor-versus-cardiac exposure, clinically achievable systemic concentrations, or cardiac safety. These findings therefore support preclinical investigation of Deslanoside; neither C4 classification nor GDI1 expression has been validated as a marker for selecting patients for this treatment.

## Methods

### Data sources and preprocessing

The discovery analysis included 584 colorectal cancer samples from the TCGA-CRC cohort that entered the molecular subtyping workflow (21). The available clinical data included overall survival, disease-specific survival, disease-free survival, progression-free survival, lymph node metastasis status, N stage, T stage, M stage, and clinical stage. Expression data were converted to gene-level values for subsequent analyses, and each cohort was normalized independently. Molecular subtype template projection was performed in TCGA-CRC, GSE12945, GSE13294, GSE14333, GSE17536, GSE26682, GSE39582, GSE41258, GSE87211, GSE92921, GSE143985, and GSE161158 (22). When multiple probes mapped to the same gene, their expression values were combined at the gene level. Subtype-specific expression patterns were displayed as within-cohort Z scores.

### Identification of lymph node metastasis-related genes and molecular subtype construction

Gene expression was compared between the LNM and nLNM groups using limma, and univariable Cox regression was performed for candidate genes with overall survival as the endpoint (23). Genes associated with LNM were selected using a Benjamini–Hochberg-adjusted P value ≤ 0.05 and an absolute limma coefficient ≥ 0.25. These genes were subsequently assessed by univariable Cox regression for overall survival, and genes with nominal P < 0.05 were retained for unsupervised subtyping. Within the discovery cohort of size 584, consensus clustering solutions with K values from 2 to 9 were compared (24). A four-cluster solution was selected after jointly considering cluster-consensus scores and consensus matrices, and the four molecular subtypes were designated C1, C2, C3, and C4. Principal component analysis was used to display subtype structure in the observed data and to compare it with a representative size-matched null dataset. UMAP was further used to visualize the expression distribution of the four subtypes (25). Within the same discovery cohort, overall survival distributions across the four molecular subtypes were described using the Kaplan-Meier method and compared using the log-rank test.

### Cross-cohort template projection and clinical composition analysis

Marker genes for the four molecular subtypes were organized into class templates, and nearest template prediction was used to assign samples from 12 cohorts to C1, C2, C3, or C4 (26). Expression data were standardized separately within each cohort, after which the within-cohort expression patterns of subtype marker genes were compared. Among TCGA-CRC samples with the corresponding clinical information, the proportions of the four molecular subtypes were calculated for the LNM and nLNM groups, different clinical stages, M stages, and T stages. Clinical composition was compared using global Pearson chi-square tests. Each comparison included samples with nonmissing annotations for the corresponding variable.

### Molecular characterization and immune-related analyses

Subtype-associated transcriptional features were defined using limma differential expression analysis that compared each molecular subtype with all remaining samples (23). Differential expression thresholds were a Benjamini–Hochberg-adjusted P value ≤ 0.05 and an absolute limma coefficient ≥ 0.5. Hallmark and KEGG gene set scores were calculated using the single-sample gene set enrichment analysis (ssGSEA) method implemented in GSVA (27–29). Signaling pathway activity was inferred using PROGENy from weighted transcriptional responses to pathway perturbations (30), and malignant functional states were scored using CancerSEA signatures (31). Somatic mutation data were arranged by molecular subtype to generate a mutation matrix, and mutation frequencies were summarized for each subtype (32). A protein association network was constructed from subtype-associated genes using STRING (33). Sample-level transcription factor activities were inferred using VIPER with human DoRothEA regulons at confidence levels A–C, and the activities of PRDM14, CTCF, and SOX2 were compared across subtypes (34, 35). Transcription factor enrichment of subtype-specific gene sets was assessed separately using ChEA3 (36). Tumor microenvironment analyses included StromalScore, ImmuneScore, ESTIMATEScore, and TumorPurity generated using ESTIMATE (37), together with predicted immunotherapy response classification and TIDE, Dysfunction, Exclusion, and CAF scores generated using TIDE (38). The immunophenoscore (IPS) and its four weighted components were calculated following the original method (39). The aggregate weighted z-score was calculated as AZ = MHC + CP + EC + SC and transformed to the 0–10 IPS scale. MHC represents antigen-processing and major histocompatibility complex-related molecules, EC effector cells, SC suppressor cells, and CP checkpoints and immunomodulators. SC includes negative weights and is not a direct cell-abundance measure. Immunoregulatory genes were organized into antigen presentation, cell adhesion, co-inhibition, co-stimulation, ligand, receptor, and other categories and were displayed in a heatmap of sample-level Z scores.

### Multi-cohort screening and clinical association analysis of GDI1

Univariable Cox regression was performed separately for candidate genes in TCGA-CRC, GSE17536, GSE39582, GSE41258, and GSE71187, followed by a summary of the cross-cohort risk direction and mean hazard ratio for each gene. GDI1 was selected for subsequent analyses according to the multi-cohort ranking. Samples in each cohort were divided according to the median GDI1 expression, and overall survival was compared using the Kaplan-Meier method and log-rank test. TCGA-CRC was also used for disease-specific survival, disease-free survival, and progression-free survival analyses and for comparisons of GDI1 expression across N stages and clinical stages. Receiver operating characteristic analysis was used to evaluate the ability of GDI1 to distinguish LNM from nLNM status in TCGA-CRC (40), and gene set enrichment analysis was performed for GDI1-associated transcriptional changes (41, 42).

### Molecular docking and molecular dynamics simulation

The AlphaFold three-dimensional structure of GDI1 corresponding to UniProt P31150 was used as the receptor (43). Missing atoms were completed, polar hydrogens were added, and the structure was converted to PDBQT format. Approved small molecules were prepared, energy-minimized, and used as ligands. Docking across the entire receptor surface was performed using AutoDock Vina 1.2.5 (44). The search box center coordinates were set to 6.114, 6.580, and 3.015 Å. The box dimensions were 75.1 × 77.3 × 74.3 Å, the grid spacing was 0.375 Å, and the exhaustiveness value was 8. A total of 9 binding modes were retained for each ligand, with an energy range of 4 kcal/mol. A total of 2990 compounds yielded complete docking results. Candidate compounds were ranked by predicted binding affinity, and Deslanoside, which tied for the highest predicted affinity in the screen, was prioritized for downstream analyses. Protein and ligand contacts in the predicted complex were analyzed using PLIP (45). Surface structure, cartoon structure, and the binding pocket were visualized using PyMOL. A 100 ns molecular dynamics simulation was performed for the predicted Deslanoside and GDI1 complex, and protein backbone root mean square deviation was used to characterize conformational changes during the simulation.

### Cell lines and culture treatments

The human colorectal cancer cell lines HCT116 and SW480 were purchased from Procell under catalog numbers CL-0096 and CL-0223, respectively. Cells were cultured in DMEM supplemented with 10% fetal bovine serum and 1% penicillin-streptomycin. DMEM, fetal bovine serum, and penicillin-streptomycin were purchased from Gibco under catalog numbers 11965092, 10099141, and 15140122, respectively. Cells were maintained at 37 °C in 5% CO_2_. Deslanoside was purchased from MedChemExpress under catalog number HY-A0154. A concentration of 5 μM Deslanoside in complete medium was used for the *in vitro* phenotypic assays. Deslanoside assays included NC, NC plus Deslanoside, shGDI1 #1 plus DMSO, and shGDI1 #1 plus Deslanoside. NC denoted empty-vector cells receiving DMSO.

### Establishment of stable GDI1 knockdown cells

Two human GDI1 shRNA target sequences were individually cloned into the pLKO.1-EGFP-Puro lentiviral vector and packaged into lentiviral particles, with the empty vector serving as NC. Following infection, cells were selected continuously with puromycin for two weeks, and GDI1 knockdown efficiency was evaluated using real-time quantitative PCR. The target sequence of shGDI1 #1 was 5′-CGCCAACTCCTGCCAAATAAT-3′, and that of shGDI1 #2 was 5′-CCCATATTTATACCCGCTCTA-3′. Both sequences were used in the *in vitro* functional assays. Stable cells expressing shGDI1 #1 were used for Deslanoside exposure and animal studies.

### RNA extraction and RT-qPCR

Total RNA was isolated using the Qiagen RNeasy Mini Kit under catalog number 74106. Complementary DNA was synthesized using the Thermo Fisher Scientific RevertAid First Strand cDNA Synthesis Kit under catalog number K1622. Real-time quantitative PCR was performed using TaKaRa TB Green Premix Ex Taq II FAST qPCR under catalog number CN830A on a BIOER LineGene 9600 Plus system. The primer pair used to detect GDI1 was purchased from OriGene under the product name qSTAR GDI1 Human qPCR Primer Pair and catalog number HP205362, corresponding to RefSeq NM_001493.3. The forward primer sequence was 5′-CCGAGACTGGAATGTTGACCTG-3′, and the reverse primer sequence was 5′-CCTTGAAGTCCAGGTAGCGAGT-3′. GAPDH was used as the reference gene, and relative GDI1 expression was calculated using the 2^−ΔΔCt^ method.

### CCK-8 assay

Cell growth-related measurements were obtained using the Cell Counting Kit-8 purchased from Dojindo under catalog number CK04. The detection reagent was added according to the manufacturer’s instructions, after which absorbance was measured at 450 nm using a SuPerMax 3200 multimode microplate reader. GDI1 knockdown experiments compared the NC, shGDI1 #1, and shGDI1 #2 groups at 0, 24, 48, 72, and 96 h after culture. For Deslanoside experiments, cells were cultured overnight after seeding before 5 μM Deslanoside or the corresponding DMSO control was added. Time 0 was defined as the start of treatment, and treatment was maintained until each measurement at 0, 24, 48, 72, and 96 h. Deslanoside experiments used the four-group design described above and were performed separately in HCT116 and SW480 cells.

### Transwell migration assay

Migration assays were performed using Corning 3422 Transwell inserts designed for 24-well plates, with a membrane diameter of 6.5 mm and a pore size of 8.0 μm. Matrigel was not used. For Deslanoside experiments, cells were pretreated with 5 μM Deslanoside or the corresponding DMSO control for 24 h before being seeded into the inserts. A total of 2 × 10^4^ cells of either HCT116 or SW480 were added to the upper chamber, and 500 μL of complete medium was added to the lower chamber. After incubation for 24 h following seeding, nonmigrated cells on the upper membrane surface were removed. Cells on the lower membrane surface were fixed with 4% paraformaldehyde purchased from Sigma-Aldrich under catalog number P6148 and subsequently stained with crystal violet from Sigma-Aldrich. Images were acquired using a CEWEI E100 microscope, and cells that had traversed the membrane were counted using ImageJ.

### Wound-healing assay

Cells from the study and control conditions were seeded in 6-well plates and cultured for 12 h in medium containing 10% fetal bovine serum before wounding. A vertical wound was created using a 200 μL pipette tip, and detached cells were removed by washing with PBS purchased from Gibco under catalog number 10010023. For Deslanoside assays, medium containing 5 μM Deslanoside or the corresponding DMSO control was added after wounding and washing, and treatment was maintained for 24 h. Wound images were acquired at 0 h and 24 h using a CEWEI E100 microscope, and wound closure was calculated using ImageJ. GDI1 knockdown assays and the four-group Deslanoside assays were performed using the same procedure in HCT116 and SW480 cells.

### Subcutaneous xenograft assay

The animal study protocol was reviewed and approved by the Animal Ethics Committee of Wuhan hualianke Biotechnology Co., Ltd. (approval no. HLK-20260116-002). The 6-week-old male BALB/c nude mice were supplied by Beijing Vital River Laboratory Animal Technology Co., Ltd. and housed under specific pathogen-free barrier conditions. HCT116 and SW480 cells stably expressing NC or shGDI1 #1 were prepared as single-cell suspensions. Each mouse received a subcutaneous injection of 3 × 10^6^ cells into the right flank, with 6 mice included in each group. GDI1 knockdown assays included NC and shGDI1 #1. For the Deslanoside study, mice were randomly assigned when the mean xenograft volume reached 50 to 100 mm³. The four groups were NC, NC plus Deslanoside, shGDI1 #1 plus DMSO, and shGDI1 #1 plus Deslanoside. NC represented empty-vector xenografts receiving DMSO. Deslanoside or DMSO was administered by intratumoral injection. Tumor volume was recorded on days 4, 8, 12, 16, 20, 24, and 28 after inoculation. Endpoint assessment was completed on day 28, when tumors were excised and weighed.

### Statistical analysis

Bioinformatic analyses were performed in R. Continuous variables were compared between two groups using the Wilcoxon rank-sum test and across multiple groups using the Kruskal-Wallis test. Pairwise comparisons between subtypes were performed using the Wilcoxon rank-sum test, and mutation frequencies were compared using Fisher’s exact test. Survival curves were estimated using the Kaplan-Meier method and compared using the log-rank test, while prognostic associations were evaluated using Cox proportional hazards regression. Multiple hypothesis testing was corrected using the Benjamini-Hochberg method where applicable. The clinical composition comparisons in **Figures 2C–F** used global Pearson chi-square tests; these exploratory comparisons are reported with unadjusted P values. Cell and animal experimental data were also organized in R and are presented as the mean and standard deviation. CCK-8 measurements and xenograft volumes were summarized as time curves at each time point. RT-qPCR data were log2-transformed and analyzed using two-sided Welch t tests for prespecified comparisons, with Holm correction applied within each cell line. Prespecified pairwise comparisons of Transwell migration, wound closure, and endpoint tumor weight were performed using two-sided Welch t tests.

## Data Availability

The datasets presented in this study can be found in online repositories. The names of the repository/repositories and accession number(s) can be found in the article/supplementary material.
